# Mental health law in Thailand

**DOI:** 10.1192/bji.2025.14

**Published:** 2025-11

**Authors:** Nacharin Phiphopthatsanee

**Affiliations:** MD, MRCPsych, MAcadMEd, Faculty of Medicine, King Mongkut’s Institute of Technology Ladkrabang, Bangkok, Thailand.

## Abstract

Thailand’s first Mental Health Act was enacted on 20 February 2008, with an amendment enforced on 15 July 2019. The Act provides a framework for mental health professionals, aiming to protect patient rights and promote societal safety by minimising the risks associated with mental illness. This article highlights key practical aspects of the legislation and discusses issues regarding its real-life application.

## Historical and cultural context

The doctor–patient relationship in Thailand has traditionally been characterised as paternalistic.^1^ Patients typically grant consent, allowing physicians to perform procedures deemed necessary without formal discussion. This dynamic remains especially prevalent in rural areas, where many patients are unaccustomed to participating in the decision-making process regarding their care. The Ministry of Public Health has introduced a standardised consent form for use in both general and psychiatric hospitals.^2^ However, patients with severe mental illnesses frequently lack the capacity to provide informed consent. Prior to the enactment of the first Mental Health Act in 2008, there was a pre-existing legislation outlining a hierarchy of surrogates for proxy consent on behalf of incapacitated individuals.^2^ Nonetheless, there was no clear legal framework governing involuntary psychiatric admissions during that period. While the Mental Health Act introduced more structured guidelines, it continues to allow relatives to consent to psychiatric admission on behalf of a patient deemed to lack decision-making capacity. This reflects the collectivist values embedded in Thai culture and their enduring influences on mental health legislation.

## Mental Health Act B.E. 2551 (2008), updated 2019^3^

### Patient rights

The Mental Health Act outlines key rights and protections for patients, including the right to receive treatment, maintain confidentiality and make decisions about their care. Physical restraint, seclusion and isolation are prohibited unless there is a significant risk to the patient or others. Additionally, electroconvulsive therapy requires either the patient’s or surrogate’s consent or, in emergencies, approval from the infirmary board. Sterilisation is permitted only with the patient’s consent.

The 2019 amendment to the Act incorporated an emphasis on rehabilitation and assigned to the director-general of the Department of Mental Health the responsibility of regulating the dissemination of any information that might cause defamation to patients.

### Admission and treatment

The Act stipulates that psychiatric treatment can be administered only after the patient has received comprehensive information and has voluntarily given consent. Psychiatric admission requires written consent from the patient. For individuals under 18 years of age or those lacking the capacity to consent, the Act allows a surrogate to provide consent on the patient’s behalf. However, the Act does not specifically provide a clear definition of decision-making capacity.

Individuals whose mental disorders place them in a ‘threatening condition’ or who ‘require treatment’ can be admitted and treated without consent. A ‘threatening condition’ is defined as behaviour posing serious harm to oneself, others or property, while ‘requirement for treatment’ refers to patients lacking the capacity to consent to treatment or admission. These involuntary admissions are restricted to certain institutions listed in the legislation. Currently, 116 government facilities, including 20 specialised psychiatric hospitals across 70 provinces, are authorised for involuntary admissions.

The Act also grants police or relevant officers, such as probation officers, the power to access any properties or vehicles to take individuals believed to be in a ‘threatening condition’ or who ‘require treatment’ to the nearest state hospital or listed institution for assessment. A search warrant is not required if there is reason to believe that an individual poses an imminent and significant risk. Mechanical restraints are prohibited unless there is a significant risk to oneself, others or property.

Upon arrival, the individual must be assessed by at least one medical doctor and one nurse within 48 h. If involuntary admission or further evaluation by an infirmary board is deemed necessary, the patient is admitted to a listed institution. A comprehensive assessment by an infirmary board – comprising a psychiatrist, a physician of any specialty, a psychiatric nurse, a lawyer and a clinical psychologist or social worker – must occur within 30 days. The board determines the treatment plan and duration of hospitalisation, which must initially not exceed 90 days. Extensions are permissible in 90-day increments. If an individual escapes from the institution, staff must coordinate with relatives and law enforcement to facilitate their return.

### Forensic section

Under the Criminal Procedure Code, if an inquiry officer or the court suspects that an alleged offender or accused individual has a severe mental illness and lacks competency to stand trial, the investigation and trial must be deferred.^4^ The individual is then conveyed to a psychiatric institution for involuntary admission. The Mental Health Act mandates that a psychiatrist at the institution must provide a diagnosis and a professional opinion regarding the person’s competency to stand trial within 45 days.

The Mental Health Act does not specify a maximum duration for admission, but requires the individual to remain hospitalised until recovery and competency to stand trial are demonstrated, unless otherwise directed by the court. The attending psychiatrist must report the patient’s progress to the inquiry officer or the court within 180 days of admission, and continue providing updates every 180 days if the individual remains hospitalised. Discharge must occur promptly following recovery and restored competency.

For first-time minor offences attributable to mental illness, the court may waive punishment and instead order the individual to undergo treatment in a psychiatric institution. In such cases, the attending psychiatrist must report the patient’s progress to the court within 180 days and provide subsequent updates every 180 days if hospitalisation continues.

### Rehabilitation

The section of the Mental Health Act addressing rehabilitation following discharge from involuntary admission is relatively brief, with substantial additions introduced in the 2019 amendment. Under this provision, the head of the infirmary is responsible for notifying the carer or relevant social care organisation to ensure continuity of care within the community. Additionally, carers are entitled to receive support to assist them in providing effective care for the patient. The Act also emphasises the involvement of relatives, community and private sectors in promoting successful reintegration into the community.

### Appeal

A patient admitted involuntarily, or their guardian, has the right to appeal the decision, which must be submitted to an appeal committee within 30 days of initiation of the section. The committee comprises the director-general of the Department of Mental Health, three patient advocates from non-governmental organisations, a psychiatrist, a clinical psychologist, a social worker, a psychiatric nurse and a lawyer. The committee is required to review the appeal and reach a decision within 30 days of its submission.

## Controversial use

In 2020, a political activist who shared a photograph of himself wearing a T-shirt with the phrase ‘I lost faith in the monarchy’ was arrested, physically restrained and subsequently admitted to a psychiatric hospital. The legal basis for this admission was not clearly communicated to the public. While some officials stated that the admission was voluntary, this claim appeared inconsistent with reports that his hands were tied with cloth during the transfer. Others suggested that the admission was based on consent provided by his relatives.^5,6^

Human rights organisations expressed concern over the detention, describing it as arbitrary. They argued that the individual did not appear to meet the criteria for involuntary detention under section, such as ‘being in a threatening condition, i.e. posing risks to himself or others’ or ‘requiring treatment, i.e. lacking decision-making capacity’, to a degree that required proxy consent or involuntary admission under the applicable legal framework. Following calls for his release and protests by human rights activists, the individual was discharged after nearly 2 weeks of hospitalisation.^5,6^

## Commentaries

Although the Thai Mental Health Act was primarily designed to safeguard patients, its emphasis on patient rights in the context of involuntary admission is limited, lacking explicit recognition of the patient’s will or the consideration of employing the ‘least restrictive method’. This reflects incompatibility with the United Nations Convention on the Rights of Persons with Disabilities (UNCRPD), to which Thailand is a signatory.^7^ Similar to mental health laws in many other countries, involuntary detention based on the diagnosis of mental illness violates Article 14 (1)b of UNCPRD, which states that ‘the existence of a disability shall in no case justify a deprivation of liberty’. Other justifications for involuntary detention, such as dangerousness, are also deemed incompatible with this Article because they are, at least partly, based on the individual’s disability, i.e. mental illness. Additionally, involuntary treatment contravenes Article 17, which maintains that ‘every person with disabilities has the right to respect for physical and mental integrity on an equal basis with others’.^8^ The provision allowing relatives or guardians to consent to a ‘voluntary’ psychiatric admission on behalf of a patient who appears to lack decision-making capacity can sometimes circumvent a proper Mental Health Act assessment, raising questions about procedural integrity. The issue is compounded by the fact that not all hospitals are authorised to admit patients under the Mental Health Act; consequently, patients who meet the criteria for involuntary admission are occasionally admitted to these facilities as ‘voluntary’ patients based on consent from relatives or guardians. Such substitute decision-making contradicts Article 12, which upholds the equal legal capacity of persons with disabilities and calls for a shift towards supported decision-making.^8^

Moreover, there is no clear framework governing patient leave of absence from the hospital. In practice, individuals admitted either voluntarily or involuntarily are typically not permitted to take leave until their discharge, further reflecting the paternalism entrenched in the system. This is also incompatible with Article 19 of UNCRPD, which upholds the right to live independently in the community and deinstitutionalisation.^8^ Furthermore, many patients and their families are unaware of their right to appeal involuntary admissions; in 2021, only one appeal was filed out of 1892 involuntary admissions nationwide.^9^

Compared with the Mental Health Act in developed countries such as the UK, Thailand’s legislation lacks mandates for the involvement of independent professionals in Mental Health Act assessments, in order to reduce the risks of overmedicalisation and unwarranted detention. Albeit uncommon, the aforementioned instance of controversial detention underscores the necessity for independent oversight. Moreover, the Act does not specify the statutory criteria of qualifications or training for doctors conducting these assessments. This may reflect the shortage of mental health professionals and resources: Thailand has just over 800 psychiatrists, who mostly work in Bangkok and major cities.^10^ Despite being included in the universal coverage scheme, mental health care remains underfunded, with only 1.8% of the health budget allocated to the Department of Mental Health in 2024.^11^

## Future direction

Public hearings were conducted in mid-2024 to discuss proposed amendments to the Mental Health Act.^12^ The draft amendments expand the definition of mental illness to encompass mental and behavioural disturbances due to substance use. Additionally, the proposals call for the establishment of a national fund to support mental health services and grant the director-general authority to regulate the dissemination of false information that could negatively affect public mental health. Nevertheless, significant challenges remain in aligning the legislation with UNCRPD and promoting a more ‘disability-neutral’ approach.

## References

[1] Wattanapisit A , Saengow U. Patients’ perspectives regarding hospital visits in the universal health coverage system of Thailand: a qualitative study. Asia Pac Fam Med 2018; 17: 9. 30186036 10.1186/s12930-018-0046-x PMC6122214

[2] Likitlersuang P. Consent in psychiatric practice. J Psychiatr Assoc Thail 1998; 43: 368–77.

[3] Department of Mental Health, Ministry of Public Health. Mental Health Act B.E. 2551 (A.D. 2008), updated in A.D. 2019 (THA). Department of Mental Health, Ministry of Public Health, 2019 (https://www.dmh-elibrary.org/items/show/705).

[4] Court of Justice. Criminal Procedure Code 2019 (THA) s 14. Court of Justice, 2019 (https://jla.coj.go.th/th/content/category/detail/id/8/cid/115/iid/121396).

[5] Reuters. *Thais Protest over Man Hospitalised after Wearing Critical T-Shirt*. Reuters, 2020 (https://www.reuters.com/article/world/thais-protest-over-man-hospitalised-after-wearing-critical-t-shirt-idUSKCN24I2C9/).

[6] Prachatai Staff. Facebook User behind Viral ‘Lost Faith’ Shirt Committed to Psychiatric Hospital. Prachatai English, 2020 (https://prachataienglish.com/node/8646).

[7] Srisuppaphon D , Sriboonroj A , Riewpaiboon W , Tangcharoensathien V. Effective implementation of the UNCRPD by Thailand State Party: challenges and potential remedies. BMC Int Health Hum Rights 2017; 17: 15. 28545526 10.1186/s12914-017-0123-5 PMC5445367

[8] Szmukler G , Daw R , Callard F. Mental health law and the UN Convention on the rights of persons with disabilities. Int J Law Psychiatry 2014; 37: 245–52. 24280316 10.1016/j.ijlp.2013.11.024 PMC4024199

[9] Office of the Secretariat to the National Mental Health Commission. *Annual Report 2021*. Office of the Secretariat to the National Mental Health Commission, 2021 (https://omhc.dmh.go.th/structure/สรุปผลการดำเนินงาน%20ลคสช%202564.pdf).

[10] Thai Health Report. *Insufficient Mental Health Professionals in Thailand*. Thai Health Report, 2023 (https://www.thaihealthreport.com/th/articles_detail.php?id=167).

[11] Department of Mental Health, Ministry of Public Health. *Annual Report 2024*. Department of Mental Health, Ministry of Public Health, 2024 (https://dmh.go.th/ebook/files/รายงานประจำปีกรมสุขภาพจิต%20ปีงบประมาณ%202567.pdf).

[12] Department of Mental Health, Ministry of Public Health. *Draft Amendments of the Mental Health Act*. Department of Mental Health, Ministry of Public Health, 2024 (https://www.law.go.th/listeningDetail?survey_id=NDA4MERHQV9MQVdfRlJPTlRFTkQ=).

